# Longitudinal changes in compliance, oxygenation and ventilatory ratio in COVID-19 versus non-COVID-19 pulmonary acute respiratory distress syndrome

**DOI:** 10.1186/s13054-021-03665-8

**Published:** 2021-07-15

**Authors:** François Beloncle, Antoine Studer, Valérie Seegers, Jean-Christophe Richard, Christophe Desprez, Nicolas Fage, Hamid Merdji, Bertrand Pavlovsky, Julie Helms, Sibylle Cunat, Satar Mortaza, Julien Demiselle, Laurent Brochard, Alain Mercat, Ferhat Meziani

**Affiliations:** 1 Medical ICU, University Hospital of Angers, Vent’Lab, University of Angers, Angers, France; 2 CNRS, INSERM 1083, MITOVASC, University of Angers, Angers, France; 3 Medical ICU, University Hospital of Strasbourg, University of Strasbourg, Strasbourg, France; 4 Oncology Data Factory and Analytics, ICO Integrated Center for Oncology, Angers, France; 5 INSERM, UMR 955 Eq 13, University of Paris-Est-Créteil, Créteil, France; 6 INSERM (French National Institute of Health and Medical Research), UMR 1260, Regenerative Nanomedicine (RNM), FMTS, Strasbourg, France; 7 Keenan Research Centre, Li Ka Shing Knowledge Institute, St. Michael’s Hospital, Toronto, Canada; 8 Interdepartmental Division of Critical Care Medicine, University of Toronto, Toronto, Canada

**Keywords:** Mechanical ventilation, Respiratory failure, Respiratory mechanics, Dead space, Acute Respiratory Distress Syndrome, Covid-19

## Abstract

**Background:**

Differences in physiology of ARDS have been described between COVID-19 and non-COVID-19 patients. This study aimed to compare initial values and longitudinal changes in respiratory system compliance (*C*_RS_), oxygenation parameters and ventilatory ratio (VR) in patients with COVID-19 and non-COVID-19 pulmonary ARDS matched on oxygenation.

**Methods:**

135 patients with COVID-19 ARDS from two centers were included in a physiological study; 767 non-COVID-19 ARDS from a clinical trial were used for the purpose of at least 1:2 matching. A propensity-matching was based on age, severity score, oxygenation, positive end-expiratory pressure (PEEP) and pulmonary cause of ARDS and allowed to include 112 COVID-19 and 198 non-COVID pulmonary ARDS.

**Results:**

The two groups were similar on initial oxygenation. COVID-19 patients had a higher body mass index, higher *C*_RS_ at day 1 (median [IQR], 35 [28–44] vs 32 [26–38] ml cmH_2_O^−1^, *p* = 0.037). At day 1, *C*_RS_ was correlated with oxygenation only in non-COVID-19 patients; 61.6% and 68.2% of COVID-19 and non-COVID-19 pulmonary ARDS were still ventilated at day 7 (*p* = 0.241). Oxygenation became lower in COVID-19 than in non-COVID-19 patients at days 3 and 7, while *C*_RS_ became similar. VR was lower at day 1 in COVID-19 than in non-COVID-19 patients but increased from day 1 to 7 only in COVID-19 patients. VR was higher at days 1, 3 and 7 in the COVID-19 patients ventilated using heat and moisture exchangers compared to heated humidifiers. After adjustment on PaO_2_/FiO_2_, PEEP and humidification device, *C*_RS_ and VR were found not different between COVID-19 and non-COVID-19 patients at day 7. Day-28 mortality did not differ between COVID-19 and non-COVID-19 patients (25.9% and 23.7%, respectively, *p* = 0.666).

**Conclusions:**

For a similar initial oxygenation, COVID-19 ARDS initially differs from classical ARDS by a higher *C*_RS_, dissociated from oxygenation. *C*_RS_ become similar for patients remaining on mechanical ventilation during the first week of evolution, but oxygenation becomes lower in COVID-19 patients.

*Trial registration*: clinicaltrials.gov NCT04385004

**Supplementary Information:**

The online version contains supplementary material available at 10.1186/s13054-021-03665-8.

## Introduction

Most of the patients admitted to ICU for coronavirus disease 2019 (COVID‐19) present severe respiratory failure fulfilling acute respiratory distress syndrome (ARDS) criteria according to the Berlin definition [1–3]. Several hypotheses emerged from the literature, but little is known about the specific pathophysiology of COVID-19-associated ARDS. Based on clinical observations reported in small series, it has been advocated that part of the patients with COVID-19 ARDS may be characterized by severe hypoxemia and relatively normal respiratory system compliance (*C*_RS_) and may beneficiate from a “less protective” ventilation compared to the “classical form” of ARDS [4]. In addition, some data suggested that COVID-19-associated ARDS may be characterized by a high pulmonary dead space fraction [5]. The description of a high ventilatory ratio (VR) which has been shown to be associated with an increased dead space in some patients with COVID-19-associated ARDS may also support this observation [6, 7]. These data may be consistent with histologic analysis of lungs from patients who died from COVID-19 showing distinctive vascular features with severe endothelial injuries and widespread thrombosis [8]. In addition, a high risk of thrombotic complications has been found in patients with COVID-19-associated ARDS [9–11]. Cumulating evidence coming from larger series tends to demonstrate that variability in clinical presentation (depending on ARDS severity) exists in COVID-19 as it has been described in non-COVID-19-associated ARDS, thus challenging this interesting conceptual “new phenotype” specific to COVID-19-associated ARDS [12–16]. Based on this statement, these authors advocated that the well-described “lung protective strategy” should be adapted to a systematic daily physiological evaluation similarly in COVID-19 and non-COVID-19-associated ARDS patients [12, 14, 16, 17]. This controversy is of clinical importance since it may impact the ventilatory approaches proposed to manage COVID-19-associated ARDS patients [18]. Facing a clinical presentation that we considered atypical, we hypothesized that the first week time course evolution of *C*_RS_ and gas exchange may differentiate COVID-19 from non-COVID-19 forms of pulmonary ARDS. The aim of this study was to prospectively assess *C*_RS_, oxygenation parameters and VR from day 1 to day 7 in patients with COVID-19-associated ARDS and to compare them to patients with pulmonary non-COVID-19-associated ARDS. For this purpose, patients admitted for COVID-19-associated ARDS were matched with patients with non-COVID-19 pulmonary ARDS included in a previously published large randomized controlled trial (*Express* Study [19]), using a propensity score matching.

## Methods

### Patients’ selection

*Patients with COVID-19-associated ARDS (COVID-19 cohort)*. Adult patients admitted from March 3 to April 27, 2020, to two French tertiary care teaching medical ICUs (University hospitals of Angers and Strasbourg, France) and intubated for COVID-19-associated ARDS, were prospectively included within 24 h after ARDS diagnosis for longitudinal physiology assessment. ARDS was defined according to the Berlin definition criteria [3]. SARS-Cov-2 infection was confirmed by real-time reverse transcriptase-polymerase chain reaction (RT-PCR) assay of nasal swabs or lower respiratory tract samples (bronchoalveolar lavage or endotracheal aspirate). Exclusion criteria were age lower than 18 years and use of extracorporeal membrane oxygenation (ECMO) within 24 h after ARDS diagnosis. Some of these patients have been included in previously published studies [9, 17].

*Patients with* pulmonary *non-COVID-19 ARDS (non-COVID-19 cohort).* Patients with non-COVID-19-associated ARDS came from the *Express* study, a large randomized control trial performed from September 2002 to December 2005, and were eligible as control patients [19]. In brief, patients with ARDS or acute lung injury using the American-European Consensus Conference on ARDS criteria [20] were enrolled in the study within 48 h after ARDS diagnosis. Patients were then randomly assigned to two different positive end-expiratory pressure (PEEP) titration strategies: PEEP was set to a level of 5 to 9 cmH_2_O in the *minimal distension strategy* or to a level set to reach a plateau pressure of 28 to 30 cmH_2_O in the *increased recruitment strategy*.

### Ventilation and sedation strategies

Both in the two centers and in the *Express* trial, recommendations for initial management included a deep sedation and the use of neuromuscular blockers for 24 to 48 h. Patients were ventilated in volume-controlled mode with a tidal volume of 6 ml kg^−1^ of predicted body weight (PBW) and a respiratory rate up to 35 min^−1^, adjusted according to arterial pH (objective between 7.30 and 7.45). The fraction of inspired oxygen (FiO_2_) was set for an arterial oxygen saturation between 88 and 98%.

In the *COVID-19 cohort*, PEEP setting was left to the discretion of attending physician according to gas exchange and hemodynamic tolerance with an upper limit of plateau pressure of 28 cmH_2_O, similar to *Express*.

All patients were switched to pressure-support ventilation when oxygenation improved and PEEP level was decreased to 5–8 cmH_2_O.

The COVID-19 patients were ventilated using a heated humidifier or a heat and moisture exchanger (HME, Humid-Vent Compact, Teleflex, Athlone, Ireland, dead space = 35 ml or Clear Therm 3 Filter, Intersurgical, Wokingham, UK, dead space = 59 ml). All the patients with non-COVID-19-associated ARDS were ventilated using a heated humidifier.

### Data collection

Day 0 was defined as the first calendar day after the onset of ARDS in the *COVID-19 cohort* or as the day of inclusion in *Express* trial in the *non-COVID-19 cohort* (mean time from the onset of ARDS to inclusion = 26.1 ± 23.1 h in *Express* [19]).

Baseline characteristics (including age, body metrics, simplified acute physiologic score II (SAPS II) [21], partial pressure of arterial oxygen (PaO_2_), FiO_2_, partial pressure of arterial carbon dioxide (PaCO_2_), set tidal volume (Vt), measured respiratory rate, measured minute ventilation, set PEEP and plateau pressure) were collected on day 0 in the two cohorts.

The type of humidification device, HME or heated humidifier was also recorded in the *COVID-19 cohort.*

The following parameters were recorded at days 1, 3 and 7 in the two cohorts (values measured from 6 to 12 am): PaO_2_, FiO_2_, PaCO_2_, set Vt, measured respiratory rate, measured minute ventilation, set PEEP and plateau pressure (measured by performing an inspiratory hold of 0.2 to 0.3 s). The use of prone positioning and inhaled nitric oxide before day 28 was also recorded.

The diagnosis of thromboembolic event (including deep venous thrombosis on Doppler Ultra Sound or acute pulmonary embolism on CT pulmonary angiography) before day 28 was recorded in the *COVID-19 cohort*.

Mortality was assessed at day 28 in the two cohorts.

### Calculated parameters

PBW was calculated using the following formula: PBW (in kg) = 50 + (0.91 × [height in cm − 152.4]) in men and PBW = 45.5 + (0.91 × [height in cm − 152.4]) in women [22].

The alveolar-arterial oxygen gradient (A-a O_2_ gradient) was estimated as follows:

A-a O_2_ gradient = ([(PB-PH_2_O) × FiO_2_) − (PaCO_2_ (mmHg)/RQ)] − PaO_2_ (mmHg)) where PB is the barometric pressure, PH_2_O the partial pressure of water and RQ the respiratory quotient. PB, PH_2_O and RQ were considered as equal to 760 mmHg, 47 mmHg and 0.8, respectively.

Estimated *C*_RS_ was computed as tidal volume divided by the difference between plateau pressure and set PEEP.

VR was computed as minute ventilation (ml/min) × PaCO_2_ (mmHg)]/(PBW (kg) × 100 × 37.5) [23].

PaO_2_/FiO_2_, A-a O_2_ gradient, *C*_RS_ and VR were calculated at days 1, 3 and 7.

### Statistical analysis

To select well-balanced subsets of patients from the COVID-19-associated ARDS cohort and non-COVID-19-associated ARDS cohort, the following covariates were identified to build a propensity-score: age, SAPS II score, PaO_2_/FiO_2_ ratio and PEEP level on day 0 [24]. The closest controls (from the non-COVID-19-associated ARDS cohort) for COVID-19 cases were identified with the smallest average absolute distance across all the matched pairs using the “optimal” method package MatchIt [24, 25]. Only controls with pulmonary non-COVID-19-associated ARDS were kept in the final analysis sample (see details in Additional file 1).

Results are presented as median [interquartile range] or number (%). Baseline characteristics and ventilatory parameters at days 1, 3 and 7 were compared between the two groups using Mann–Whitney test for quantitative variables and Chi-square test for categorical variables. A Wilcoxon signed rank test was used to compare variables between day 1 and day 7. Correlations between ventilatory parameters were assessed using Spearman test. To identify variables associated with *C*_RS_ and VR at day 1 and day 7 successively, multiple linear regression models were built separately for *C*_RS_ and VR-dependent variables, including COVID-19 diagnosis, set PEEP, PaO_2_/FiO_2_ ratio as independent variables and additionally humidification device for VR. These independent variables were predefined based on a physiological reasoning. The mortality at day 28 was compared between the patients with VR at day 1 lower or higher than 2 in the patients with COVID-19 and in those with pulmonary non-COVID-19-associated ARDS.

All tests were performed with a type I error set at 0.05. The statistical analysis was performed using R version 3.6.2 (R Core Team (2019), R: a language and environment for statistical computing, R Foundation for Statistical Computing, Vienna, Austria. URL https://www.R-project.org/.) and Prism (GraphPad Software v5.0b, La Jolla, CA, USA).

## Results

### Flow chart, patients characteristics and ventilatory parameters at inclusion

One hundred and thirty-five patients with COVID-19-associated ARDS and 767 patients with non-COVID-19-associated ARDS were, respectively, included. One hundred and twelve patients with COVID-19-associated ARDS could be matched with 198 patients with pulmonary non-COVID-19-associated ARDS and were enrolled in the study (Additional file 2: Fig. S1).

The main characteristics and ventilatory parameters at inclusion of the matched patients are described in Table 1.

**Table 1:** Characteristics and ventilatory parameters at inclusion of the matched patients with COVID-19 and pulmonary non-COVID-19 ARDS

	COVID-19 patients *n* = 112	Pulmonary non-COVID-19 ARDS patients *n* = 198	*p* value
Age, years	63 [51–72]	60 [48–71]	0.196
SAPS II	47 [37–58]	48 [37–59]	0.562
Female sex, n	36 (32)	50 (25)	0.193
BMI, kg m^−2^	29 [26–33]	26 [23–29]	< 0.001
ARDS severity, n			
Mild	20 (18)	51 (26)	0.112
Moderate	66 (59)	117 (59)	0.978
Severe	26 (23)	30 (15)	0.706
Tidal volume, ml kg^−1^ PBW	6.1 [5.9–6.9]	6.9 [6.1–8.1]	< 0.001
Respiratory rate, cycles min^−1^	27 [25–30]	30 [25–35]	< 0.001
Volume minute, L min^−1^	10.5 [9.3–11.8]	11.7 [10–14]	< 0.001
PaCO_2_, mmHg	38 [34–43]	43 [37–49]	< 0.001
PEEP set, cmH_2_O	12 [10–14]	10 [9–12]	0.072
Plateau pressure, cmH_2_O	24 [20–27]	25 [22–28]	0.655
Respiratory system compliance, ml cmH_2_O^−1^	35 [28–43]	28 [23–36]	< 0.001
PaO_2_/FiO_2_, mmHg	143 [103–184]	134 [98–178]	0.404
A-a O_2_ gradient, mmHg	347 [242–514]	351 [271–485]	0.554
Ventilatory ratio	1.5 [1.3–2.0]	2.0 [1.6–2.6]	< 0.001
Cause of lung injury, n			
COVID-19	112 (100)	–	–
Pneumonia	–	151 (76)	–
Aspiration	–	47 (24)	–

SAPS II, simplified acute physiology score II; BMI, body mass index; PBW, predicted body weight; PEEP, positive end expiratory pressure; PaO_2_, partial pressure of arterial oxygen; FiO_2_, fraction of inspired oxygen; A-a O_2_ gradient, alveolar-arterial oxygen gradient; PaCO_2_, partial pressure of arterial carbon dioxide. Results are presented as median [interquartile range] or number (%)

The main characteristics and ventilatory parameters at inclusion of all the patients included in the two cohorts before matching are available in Additional file 2: Table S1.

### Ventilatory parameters at day 1, day 3 and day 7 in patients with COVID-19 and non-COVID-19-associated ARDS

**Fig. 1:**
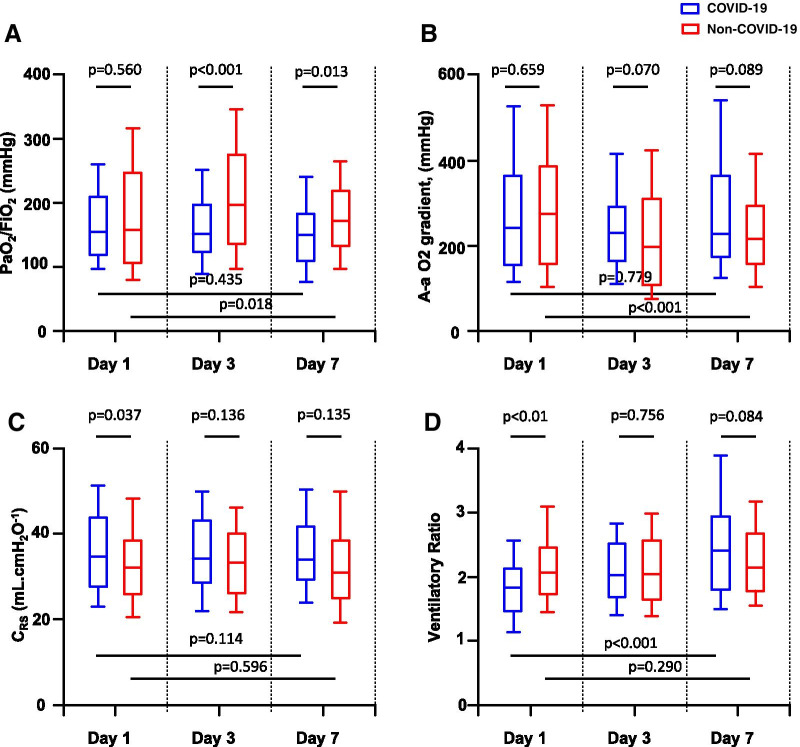
Ratio of partial pressure of arterial oxygen (PaO_2_) over fraction of inspired oxygen (FiO_2_) (**A**), alveolar-arterial oxygen gradient (A-a O_2_ gradient) (**B**), compliance of the respiratory system (*C*_RS_) (**C**) and ventilatory ratio (**D**) in matched patients with COVID-19 and non-COVID-19 pulmonary acute respiratory distress syndrome at day 1, day 3 and day 7. Boxplots display medians, 10th, 25th, 75th, and 90th percentiles

Ventilatory parameters changes in matched COVID-19-associated ARDS and pulmonary non-COVID-19-associated ARDS at day 1, day 3 and day 7 are presented in Table 2 and Fig. 1. Prone positioning was used in 61 (54%) and 44 (22%) matched patients with COVID-19 and non-COVID-19-associated ARDS, respectively (*p* < 0.001) and inhaled nitric oxide was used in 14 (13%) and 57 (29%) matched patients with COVID-19 and non-COVID-19-associated ARDS, respectively (*p* = 0.001).

**Table 2:** Ventilatory parameters in matched patients with COVID-19 (*n* = 112) and non-COVID-19 pulmonary ARDS (*n* = 198) at days 1, 3 and 7

	Day 1			Day 3			Day 7		
	COVID-19	Non-COVID-19	*p* value	COVID-19	Non-COVID-19	*p* value	COVID-19	Non-COVID-19	*p* value
Extubated, n	0 (0)	3 (2)	0.556	5 (4)	14 (7)	0.463	27 (24)	38 (19)	0.313
On PSV, n	11 (10)	0 (0)	< 0.001	25 (22)	5 (3)	< 0.001	31 (28)	16 (9)	< 0.001
On ACV, n	97 (87)	186 (93)	0.062	75 (68)	162 (82)	0.005	38 (34)	119 (60)	< 0.001
On ECMO, n	2 (2)	0 (0)	0.130	3 (3)	0 (0)	0.046	7 (6)	0 (0)	< 0.001
Tidal volume, ml kg PBW^−1^	6.1 [5.9–6.8]	6.0 [6.0–6.0]	0.014	6.1 [5.9–6.9]	6.0 [6.0–6.1]	0.190	6.4 [5.9–7.4]	6.0 [6.0–6.8]	0.572
Respiratory rate, min^−1^	27 [24–30]	30 [26–34]	< 0.001	28 [25–33]	29 [24–33]	0.884	31 [26–35]	26 [20–32]	0.007
Minute Ventilation, L min^−1^	10.9 [9.3–12.6]	11.9 [9.8–13.0]	0.059	11.5 [10.3–14.2]	11.6 [10.0–13.2]	0.553	12.3 [10.4–14.6]	12.5 [10.4–14.0]	0.954
PEEP set, cmH_2_O	12 [10–14]	12 [8–16]	0.744	12 [9–14]	10 [7–16]	0.489	14 [10–15]	7 [5–10]	< 0.001
Plateau pressure, cmH_2_O	24 [21–28]	27 [22–28]	0.029	25 [21–28]	26 [20–28]	0.832	27 [23–28]	23 [19–28]	0.017
FiO_2_	0.5 [0.4–0.7]	0.6 [0.5–0.8]	0.021	0.5 [0.4–0.6]	0.5 [0.3–0.7]	0.426	0.5 [0.4–0.7]	0.5 [0.4–0.6]	0.649
pH	7.39 [7.33–7.45]	7.36 [7.30–7.41]	< 0.001	7.38 [7.33–7.43]	7.40 [7.35–7.44]	0.109	7.41 [7.34–7.43]	7.43 [7.37–7.48]	0.001
PaCO_2_, mmHg	42 [36–46]	43 [39–50]	0.030	44 [40–49]	42 [37–50]	0.163	48 [41–55]	42 [36–48]	< 0.001

PSV, pressure support ventilation; ACV, assisted controlled ventilation; ECMO, extracorporeal membrane oxygenation; PBW, predicted body weight; PEEP, positive end-expiratory pressure; FiO_2_, fraction of inspired oxygen; PaCO_2_, partial pressure of arterial carbon dioxide. Results are presented as median [interquartile range] or number (%)

PaO_2_/FiO_2_ ratio was not different between the two patients’ groups at day 1 but became higher in the patients with non-COVID-19-associated ARDS than in those with COVID-19-associated ARDS at day 3 and day 7 (Fig. 1A). PaO_2_/FiO_2_ ratio was higher at day 7 than at day 1 in the patients with pulmonary non-COVID-19-associated ARDS but not in those with COVID-19-associated ARDS. A-a O_2_ gradient was not different between the two patients’ groups at day 1, day 3 and day 7 but became lower at day 7 compared to day 1 only in the patients with non-COVID-19-associated ARDS (Fig. 1B).

*C*_RS_ was higher in the COVID-19 patients than in the control non-COVID-19 patients at day 1 and but not at days 3 and 7 (Fig. 1C).

VR was lower in the patients with COVID-19-associated ARDS than in the patients with non-COVID-19-associated ARDS at day 1 but was not different between the two groups at days 3 and 7 (Fig. 1D). VR significantly increased from day 1 to day 7 only in patients with COVID-19-associated ARDS.

### Relationship between PaO_2_/FiO_2_ ratio and ***C***_RS_ or VR in patients with COVID-19 and non-COVID-19-associated ARDS

**Fig. 2:**
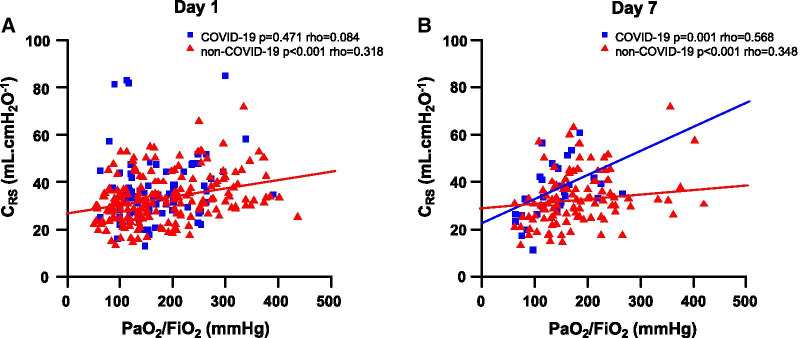
Respective correlations between the ratio of partial pressure of arterial oxygen (PaO_2_) over fraction of inspired oxygen (FiO_2_) and the compliance of the respiratory system (*C*_RS_) at day 1 (**A**) and day 7 (**B**) in the matched patients with COVID-19 and non-COVID-19 pulmonary ARDS

At day 1, *C*_RS_ was positively correlated with the PaO_2_/FiO_2_ ratio in the patients with pulmonary non-COVID-19-associated ARDS, but not in those with COVID-19-associated ARDS (Fig. 2A). These two parameters were correlated in the two groups of patients at day 7 (Fig. 2B).

VR was negatively correlated with the PaO_2_/FiO_2_ ratio in the patients with COVID-19 and in those with pulmonary non-COVID-19-associated ARDS at day 1 and day 7 (Additional file 2: Fig. S2A and Fig. S2B).

### Determinants of ***C***_RS_ and VR from day 1 to day 7 in patients with COVID-19 and non-COVID-19-associated ARDS

#### Changes in VR from day 1 to day 7 in patients with COVID-19-associated ARDS according to the humidification device

**Fig. 3:**
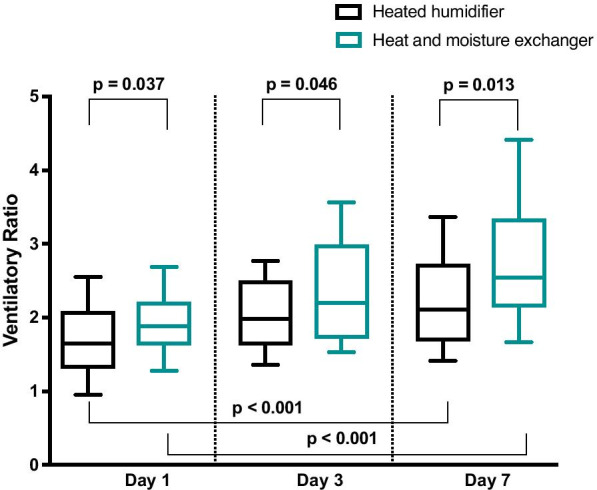
Ventilatory ratio at day 1, day 3 and day 7 in patients with COVID-19-associated acute respiratory distress syndrome ventilated using a heat and moister exchanger or a heated humidifier. Boxplots display medians, 10th, 25th, 75th, and 90th percentiles

Among the 112 matched patients with COVID-19-associated ARDS, 60 (54%) were ventilated using an HME and 52 (46%) were ventilated using a heated humidifier. VR was higher at days 1, 3 and 7 in the COVID-19 patients ventilated using an HME than in those ventilated using a heated humidifier (Fig. 3). In patients with COVID-19-associated ARDS, VR increased from day 1 to day 7 whatever the used humification device. Minute ventilation, PaCO_2,_ pH and PEEP levels in the COVID-19 patients ventilated using a HME and in those ventilated using a heated humidifier are presented in Additional file 2: Table S2.

VR was higher at day 1 in control non-COVID-19 patients (all ventilated using a heated humidifier) than in COVID-19 patients ventilated using a heated humidifier (*p* < 0.001). VR was not different at days 3 and 7 between COVID-19 patients ventilated using a heated humidifier and control non-COVID-19 patients (*p* = 0.270 and *p* = 0.746, respectively).

#### Changes in VR from day 1 to day 7 in patients with COVID-19-associated ARDS according to the presence or absence of thromboembolic event

Among the 112 matched patients with COVID-19-associated ARDS, a thromboembolic event was diagnosed in 23 patients (21%). Among these 23 patients, a pulmonary embolism was diagnosed in 19 patients. VR was higher at days 1, 3 and 7 in the COVID-19 patients with a thromboembolic event than in those without (Additional file 2: Fig. S3). VR increased from day 1 to day 7 in the patients with COVID-19-associated ARDS with or without thromboembolic event.

#### Multivariate analyses

In multivariate regression analyses, after adjustment on PaO_2_/FiO_2_ ratio and set PEEP, *C*_RS_ was significantly higher in COVID-19 patients than in non-COVID-19 patients at day 1, but was not significantly different at day 7, Table 3A. The PaO_2_/FiO_2_ ratio and the PEEP level were associated with *C*_RS_ at day 1 and day 7.

**Table 3:** Results of multivariate analyses for prediction of respiratory system compliance (A) and ventilatory ratio (B) at days 1 and 7

	*C*_RS_ at day 1			*C*_RS_ at day 7		
	Estimate	Std. Error	*p* value	Estimate	Std. Error	*p* value
(A)						
COVID-19 versus non-COVID-19	4.250	1.512	0.005	2.460	3.224	0.447
PEEP	0.726	0.180	< 0.001	0.671	0.300	0.027
PaO_2_/FiO_2_	0.020	0.009	0.020	0.057	0.018	0.002

	VR at day 1			VR at day 7		
	Estimate	Std. Error	*p* value	Estimate	Std. Error	*p* value
(B)						
COVID-19 versus non-COVID-19	− 0.691	0.117	< 0.001	− 0.135	0.263	0.608
PEEP	0.022	0.009	0.021	0.051	0.015	0.001
PaO_2_/FiO_2_	− 0.002	0.001	< 0.001	− 0.004	0.001	< 0.001
Heated humidifier versus HME	− 0.468	0.136	0.001	− 0.374	0.279	0.182

*C*_RS_, respiratory system compliance; PEEP, positive end-expiratory pressure; VR, ventilatory ratio; PaO_2_, partial pressure of arterial oxygen; FiO_2_, fraction of inspired oxygen; HME, heat and moisture exchanger

In multivariate regression analyses, after adjustment on PaO_2_/FiO_2_ ratio, set PEEP and humification device, VR was significantly lower in COVID-19 patients than in non-COVID-19 patients at day 1, but was not significantly different at day 7, Table 3B. The PaO_2_/FiO_2_ ratio and the PEEP level were associated with VR at day 1 and day 7. The use of heated humidifier was significantly associated with lower VR than the use of HME at day 1 but not at day 7.

### Outcomes

There was no difference in overall mortality at day 28 between the patients with COVID-19-associated ARDS and the control patients with pulmonary non-COVID-19-associated ARDS (25.9% and 23.7%, respectively, *p* = 0.666).

There was no difference in overall mortality at day 28 between the patients with VR at day 1 higher or lower than 2 in the patients with COVID-19-associated ARDS and in those with pulmonary non-COVID-19-associated ARDS (Additional file 2: Table S3).

## Discussion

The main observations of the present matched cohort study could be summarized as follows:

1. COVID-19-associated ARDS patients exhibited significantly higher *C*_RS_ at day 1 than pulmonary non-COVID-19-associated ARDS patients, matched on age, SAPS II, PaO_2_/FiO_2_ ratio and PEEP level. At day 7, *C*_RS_ did not differ between groups but hypoxemia was more profound in COVID-19 patients, suggesting the persistence of a possible dissociation between hypoxemia and respiratory mechanics.
2. Oxygenation and *C*_RS_ were positively correlated at day 1 in non-COVID-19, but not in COVID-19-associated ARDS. These parameters were positively correlated in the two groups of patients at day 7.
3. By contrast with our expectation, COVID-19-associated ARDS patients exhibited lower VR at day 1 compared to non-COVID-19 patients. VR of COVID-19-associated ARDS significantly increased during the first week of evolution and tended to be higher at day 7 in COVID-19 than in non-COVID-19 patients.
4. Multivariate analyses showed that differences in *C*_RS_ and VR observed between COVID-19 and pulmonary non-COVID-19-associated ARDS at day 1 no longer existed at day 7 after adjustment on PEEP level, PaO_2_/FiO_2_ ratio and humidification device.

Previously published studies assessing respiratory mechanics of COVID-19-associated ARDS showed heterogenous results [12–16]. The present study is the first to compare the evolution of respiratory mechanics in COVID-19 and non-COVID-19 pulmonary ARDS over a seven-day period. The slightly but significantly higher *C*_RS_ measured at day 1 in COVID-19 compared to non-COVID-19 patients is consistent with previous observations [14, 16]. Some authors suggested that a relatively high compliance associated with low PaO_2_/FiO_2_ ratio may characterize a phenotype subgroup of COVID-19-associated ARDS patients that deserves a specific ventilatory approach [26]. On the contrary, others advocated that “this phenotype” is simply a clinical form also observed in some non-COVID-19 ARDS patients that depends on severity and evolution [27, 28]. The present observations suggest that initial differences characterizing COVID-19 ARDS do not exist anymore at day 7.

Ventilatory ratio was lower at day 1 in patients with COVID-19, but an increase over time was observed in these patients, whereas it was not observed in control non-COVID-19 patients. The results of the multivariate analysis showing no statistical difference in VR at day 7 between COVID-19 and non-COVID-19 patients do not support that this increase in VR observed in COVID-19-associated ARDS could only reflect the “pulmonary vascular alteration”.

The significantly higher level of set PEEP at day 7 reported in COVID-19-associated ARDS may have directly impacted VR and *C*_RS_ changes since high PEEP levels may lead to overdistension and increase alveolar dead space. This difference may be explained by several differences concerning the initial unusual clinical presentation of COVID 19-ARDS as well as the ventilation strategies since more than 10 years separate the two cohorts. As a result, physiological observations performed in the present study might reflect more differences in management strategies rather than differences in pathophysiology. Thus, the multivariate analysis showed that no difference in VR or *C*_RS_ was observed between COVID-19 and non-COVID-19 patients after adjustment on PEEP level.

Importantly, in patients with COVID-19-associated ARDS, the substantially lower VR observed in the subgroup of patients ventilated with a heated humidifier suggests that a large part of the increase in VR may be related to the additional instrumental dead space induced by the HME filter as previously described [29]. The humidification device is thus an important determinant of dead space that has not been specifically considered in previous studies reporting increased VR in COVID-19 patients [7]. The impact of the increased instrumental dead space on PaCO_2_ depends on respiratory rate and Vt combinations [30]. Thus, the lower respiratory rate and slightly higher Vt might have contributed to the lower VR observed in COVID-19 patients at day 1.

The difference observed at day 1 between COVID-19 and non-COVID-19 patients is consistent with the description of patients having a higher compliance for the same level of oxygenation. In addition, our observations show that the natural course of evolution after intubation tends to erase the differences in compliance but that COVID-19 patients become more hypoxemic, again suggesting a dissociation between hypoxemia and compliance. Recent data suggest that tidal volume reduction is mostly beneficial for patients with low compliance, and our data are therefore important in this context [31]. Patients’ management must be individually adapted to the disease severity and the physiological measurements of driving pressure and compliance rather than the initial presentation.

Our study has several limitations. First, the number of patients included in the analysis is relatively small despite the two centers design of the study. Second, as discussed above, we cannot exclude that part of the ventilation strategies not considered in the analysis have changed between the two cohorts. We observed differences in the use of prone positioning, and inhaled nitric oxide between COVID-19 and non-COVID-19 patients. Differences in non-invasive oxygenation strategies before intubation may also have impacted the results. And changes in sedation level and neuromuscular blockers use may be associated with changes in CO_2_ production and thus with PaCO_2_ and VR. Third, we limited the analysis to patients with different causes of pulmonary ARDS since it was not possible to identify patients with “pure” viral pneumonia in the *non-COVID-19 cohort*. Fourth, although VR has been reported in several studies and its reliability is well accepted, VR is definitively different than a direct measurement of alveolar dead space, which requires a cumbersome technique rarely used in clinical studies [6, 32]. In addition, total PEEP was not systemically monitored and set PEEP was used to calculate *C*_RS_. Furthermore, recruitment induced by PEEP was not assessed in the present study, while it has been shown to change over time [17]. Lastly, thromboembolic events were not collected in the *Express* study. In the *COVID-19 cohort*, thromboembolic events were confirmed based on pulmonary CT and/or ultrasound in patients exhibiting clinical suspicion. And despite their potential interest for the diagnosis of thromboembolic events, D-dimer values were not systematically monitored in COVID-19 patients [33].

## Conclusion

Patients with COVID-19-associated ARDS exhibited at day 1 a slightly but significantly higher compliance dissociated from oxygenation and a lower VR compared to patients with non-COVID-19 pulmonary ARDS. VR significantly increased during the first week of evolution in COVID-19 but not in non-COVID-19 patients. Differences observed at day 1 were no longer existing at day 7 after adjustment on PEEP, PaO_2_/FiO_2_ ratio and humidification device. The present findings suggest that specific features of COVID-19 ARDS observed at day 1 disappeared during the first week of evolution due to the natural course of ARDS and the differences in ventilatory management compared to non-COVID-19 pulmonary ARDS.

## Supplementary Information

**Additional file 1**: Case-control selection

**Additional file 2**: Supplementary tables and figures

## Data Availability

The datasets analyzed during the current study are available from the corresponding author on reasonable request.

## Abbreviations

A-a O_2_ gradient: Alveolar-arterial oxygen gradient
ARDS: Acute Respiratory Distress Syndrome
*C*_RS_: Respiratory system compliance
FiO_2_: Fraction of inspired oxygen
HME: Heat and moisture exchanger
PaCO_2_: Partial pressure of arterial carbon dioxide
PaO_2_: Partial pressure of arterial oxygen
PBW: Predicted body weight
PEEP: Positive end-expiratory pressure
SAPS II: Simplified acute physiologic score II
Vt: Tidal volume
VR: Ventilatory ratio
