# Supplementary material for: Longitudinal changes in compliance, oxygenation and ventilatory ratio in COVID-19 versus non-COVID-19 pulmonary acute respiratory distress syndrome
Source: Crit Care. 2021 Jul 15;25:248. doi: 10.1186/s13054-021-03665-8 (PMC8280689; doi:10.1186/s13054-021-03665-8)
Supplement: Supplementary file 1 — Additional file 1: Case-control selection [file 13054_2021_3665_MOESM1_ESM.docx]

**Additional File 1. Case-control selection.**

**Longitudinal changes in Compliance, Oxygenation and Ventilatory Ratio in COVID-19 *vs.* non-COVID-19 associated Acute Respiratory Distress Syndrome.**

François Beloncle, Antoine Studer, Valérie Seegers, Jean-Christophe Richard, Christophe Desprez, Nicolas Fage, Hamid Merdji, Bertrand Pavlovsky, Julie Helms, Sibylle Cunat, Satar Mortaza, Julien Demiselle, Laurent Brochard, Alain Mercat, Ferhat Meziani.

**Case-control selection**

-Step 1: to select well-balanced subsets of patients from the COVID-19 associated ARDS cohort and non-COVID-19 associated ARDS cohort, a set of pretreatment control variables was identified to build the propensity-score: age, SAPS II score, PaO_2_/FiO_2_ ratio and PEEP level on day 0 [1] .

The closest controls (from the non-COVID-19 associated ARDS cohort) for each COVID-19 case unit were identified with the smallest average absolute distance across all the matched pairs using the “optimal” methodpackage MatchIt [1,2].

Summaries for all data, match data and percent balance improvement and graphical representations of QQ plots and distribution of propensity scores are presented below.

-Step 2: as a statistical difference subsists in PEEP levels at inclusion between the COVID-19 and non-COVID-19 patients, a maximum absolute difference of set PEEP at inclusion of 2 cmH_2_O between the two groups of patients was decided to keep the matched control patients in the final analysed sample.

Each COVID-19 patient should have at least one control non-COVID-19 patient or was removed from final analysed sample

-Step 3: control patients with extra-pulmonary ARDS were excluded from the analysis: only patients with pulmonary ARDS were kept as control patients in the final analysis.

-Step 4: one Covid-19 patient and the corresponding control patient were removed from the analysis because of missing data.

**Summary of balance for all data:**

| **Before matching** | | **Means.treated** | | **Means.control** | | **SD.control** | | | **Mean.diff** | | |  |
| --- | --- | --- | --- | --- | --- | --- | --- | --- | --- | --- | --- | --- |
| **Age** | | 61.46 | | 59.42 | | | 15.43 | | | 2.04 | | |
| **SAPS II** | | 38.06 | | 41.06 | | | 16.91 | | | -3.00 | | |
| **PaO_2_/FiO_2_ ratio** | | 146.82 | | 142.89 | | | 56.46 | | | 3.93 | | |
| **Set PEEP** | | 11.33 | | 8.06 | | | 3.53 | | | 3.26 | | |
|  |  | |  | |  | | |  | | |  |  |
| **After matching** | | **Means.treated** | | **Means.control** | | | **SD.control** | | **Mean.diff** | | | |
| **Age** | | 61.45 | | 59.51 | | | 13.88 | | 1.94 | | | |
| **SAPS II** | | 37.54 | | 39.01 | | | 13.89 | | -1.47 | | | |
| **PaO_2_/FiO_2_ ratio** | | 146.12 | | 142.61 | | | 55.61 | | 3.51 | | | |
| **Set PEEP** | | 11.25 | | 10.60 | | | 3.20 | | 0.65 | | | |

**References**

1. Gu XS, Rosenbaum PR. Comparison of Multivariate Matching Methods: Structures, Distances, and Algorithms. J Comput Graph Stat. 1993;2:405–20.

2. Ho D, Imai K, King G, Stuart EA. MatchIt: Nonparametric Preprocessing for Parametric Causal Inference. J Stat Softw. 2011;42:1–28.
