# Supplementary material for: Longitudinal changes in compliance, oxygenation and ventilatory ratio in COVID-19 versus non-COVID-19 pulmonary acute respiratory distress syndrome
Source: Crit Care. 2021 Jul 15;25:248. doi: 10.1186/s13054-021-03665-8 (PMC8280689; doi:10.1186/s13054-021-03665-8)
Supplement: Supplementary file 2 — Additional file 2: Supplementary tables and figures [file 13054_2021_3665_MOESM2_ESM.docx]

**Additional file 2: supplementary tables and figures**

**Longitudinal changes in Compliance, Oxygenation and Ventilatory Ratio in COVID-19 *vs.* non-COVID-19 associated Acute Respiratory Distress Syndrome.**

François Beloncle, Antoine Studer, Valérie Seegers, Jean-Christophe Richard, Christophe Desprez, Nicolas Fage, Hamid Merdji, Bertrand Pavlovsky, Julie Helms, Sibylle Cunat, Satar Mortaza, Julien Demiselle, Laurent Brochard, Alain Mercat, Ferhat Meziani.

**Table S1. Patients’ characteristics and ventilatory parameters at inclusion of all patients in COVID-19 and non-COVID-19 cohorts before matching.**

|  | ***COVID-19* cohort before matching**  **n=135** | ***Non-COVID-19* cohort**  **before matching**  **n=767** | ***p* value** |
| --- | --- | --- | --- |
| **Age, years** | 63 [51-71] | 61 [49-72] | 0.51 |
| **SAPS II** | 48 [37-61] | 50 [38-62] | 0.22 |
| **Female sex, n** | 40 [30] | 251 [33] | 0.55 |
| **BMI, Kg.m^-2^** | 29 [26-33] | 26 [23-29] | <0.001 |
| **ARDS severity, n**  Mild  Moderate  Severe | 30 (22)  74 (55)  31 (23) | 121 (16)  442 (57)  204 (27) | 0.08  0.57  0.40 |
| **Tidal volume, ml.kg^-1^ PBW** | 6.2 [5.9-6.8] | 6.0 [6.0-6.0] | <0.001 |
| **Respiratory rate, cycles.min^-1^** | 27 [25-30] | 29 [25-35] | <0.001 |
| **Volume minute, L.min^-1^** | 10.5 [9.3-12.1] | 11.0 [9.2-13.1] | 0.04 |
| **PaCO_2_, mmHg** | 38 [33-44] | 40 [35-47] | 0.02 |
| **PEEP set, cmH_2_O** | 12 [10-14] | 9 [6-12] | <0.001 |
| **Plateau pressure, cmH_2_O** | 24 [20-27] | 23 [20-27] | 0.48 |
| **Respiratory system compliance, ml.cmH_2_O^-1^** | 36 [29-44] | 29 [22-38] | <0.001 |
| **PaO_2_/FiO_2_, mmHg** | 146 [104-193] | 138 [98-180] | 0.1 |
| **A-a O_2_ gradient, mmHg** | 335 [237-513] | 324 [244-485] | 0.72 |
| **Ventilatory Ratio** | 1.6 [1.3-2.0] | 1.9 [1.6-2.4] | <0.001 |
| **Cause of lung injury, n** |  |  |  |
| Pneumonia | 135 (100) | 392 (51) | <0.001 |
| Aspiration | 0 (0) | 164 (21) | - |
| Intra-abdominal sepsis | 0 (0) | 60 (8) | - |
| Other sepsis | 0 (0) | 39 (5) | - |
| Acute pancreatitis | 0 (0) | 22 (3) | - |
| Other | 0 (0) | 90 (12) | - |

SAPS II, simplified acute physiology score II; BMI, body mass index; PBW, predicted body weight; PEEP, positive end expiratory pressure; PaO_2_, partial pressure of arterial oxygen; FiO_2_, fraction of inspired oxygen; A-a O_2_ gradient, alveolar-arterial oxygen gradient; PaCO_2_, partial pressure of arterial carbon dioxide. Results are presented as median [interquartile range] or number (%)

**Table S2. Ventilatory parameters in matched patients with COVID-19 associated ARDS ventilated using a heated humidifier or a heat and moisture exchanger at days 1, 3 and 7.**

|  | **Day 1** | | |  | **Day 3** | | |  | **Day 7** | | |
| --- | --- | --- | --- | --- | --- | --- | --- | --- | --- | --- | --- |
|  | **Heated Humidifier** | **HME** | ***p value*** |  | **Heated Humidifier** | **HME** | ***p value*** |  | **Heated Humidifier** | **HME** | ***p value*** |
| **PaCO_2_, mmHg** | 42 [37-48] | 43 [39-48] | 0.286 |  | 42 [37-48] | 46 [42-52] | <0.001 |  | 42 [36-49] | 50 [44-56] | <0.001 |
| **pH** | 7.37 [7.31-7.42] | 7.39 [7.34-7.44] | 0.099 |  | 7.40 [7.35-7.44] | 7.38 [7.33-7.42] | 0.076 |  | 7.43 [7.37-7.49] | 7.41 [7.32-7.44] | 0.017 |
| **Minute Ventilation, L .min^-1^** | 11.3 [9.7-13.2] | 11.2 [9.3-13.3] | 0.858 |  | 11.4 [10.0-13.8] | 11.5 [10.1-14.4] | 0.475 |  | 12.3 [10.3-41.0] | 13.1 [10.4-15.4] | 0.256 |
| **PEEP set, cmH_2_O** | 12 [8-15] | 12 [8-14] | 0.358 |  | 11 [8-16] | 11 [9-12] | 0.910 |  | 7 [5-12] | 12 [9-14] | <0.001 |

HME, heat and moisture exchanger; PaCO_2_, partial pressure of arterial carbon dioxide; PEEP, positive end-expiratory pressure. Results are presented as median [interquartile range].

**Table S3. Mortality at day 28 in patients with COVID-19 and pulmonary non-COVID-19 associated ARDS according to ventilatory ratio. *p=0.267***

|  | ***COVID-19*** | ***Non- COVID-19*** |
| --- | --- | --- |
| **Ventilatory Ratio < 2** | 15.5% | 21.3% |
| **Ventilatory Ratio ≥ 2** | 30% | 25.7% |

**
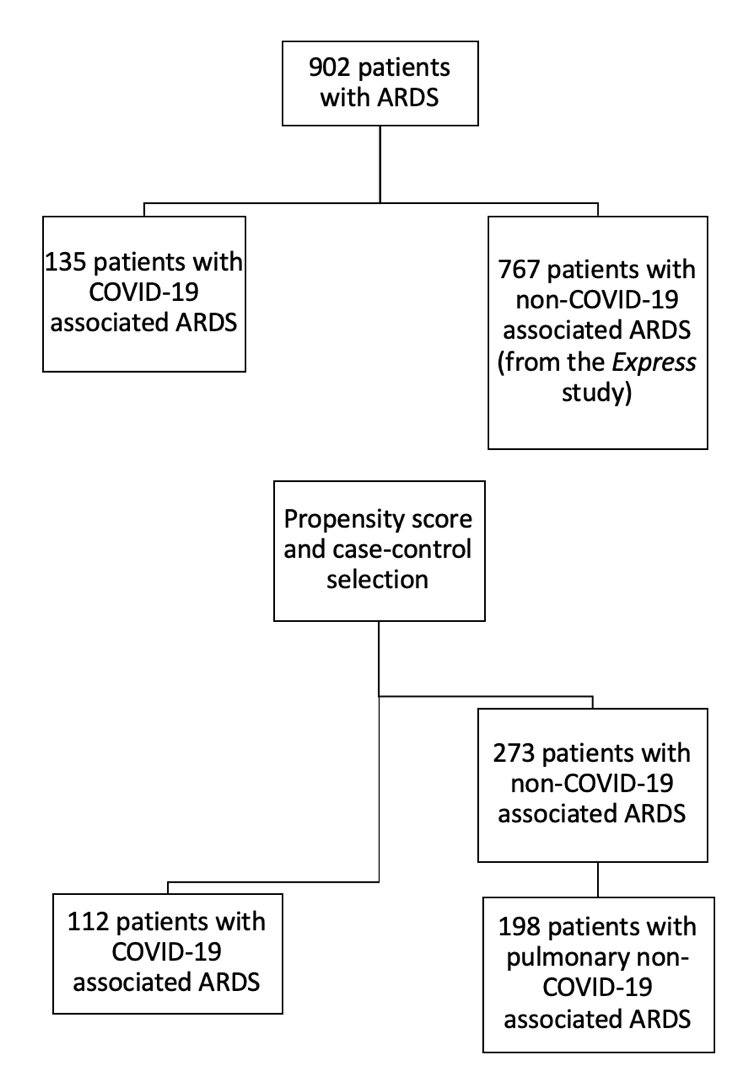
**

**Fig. S1** Flow chart. ARDS, Acute Respiratory Distress Syndrome.

**A B**

**
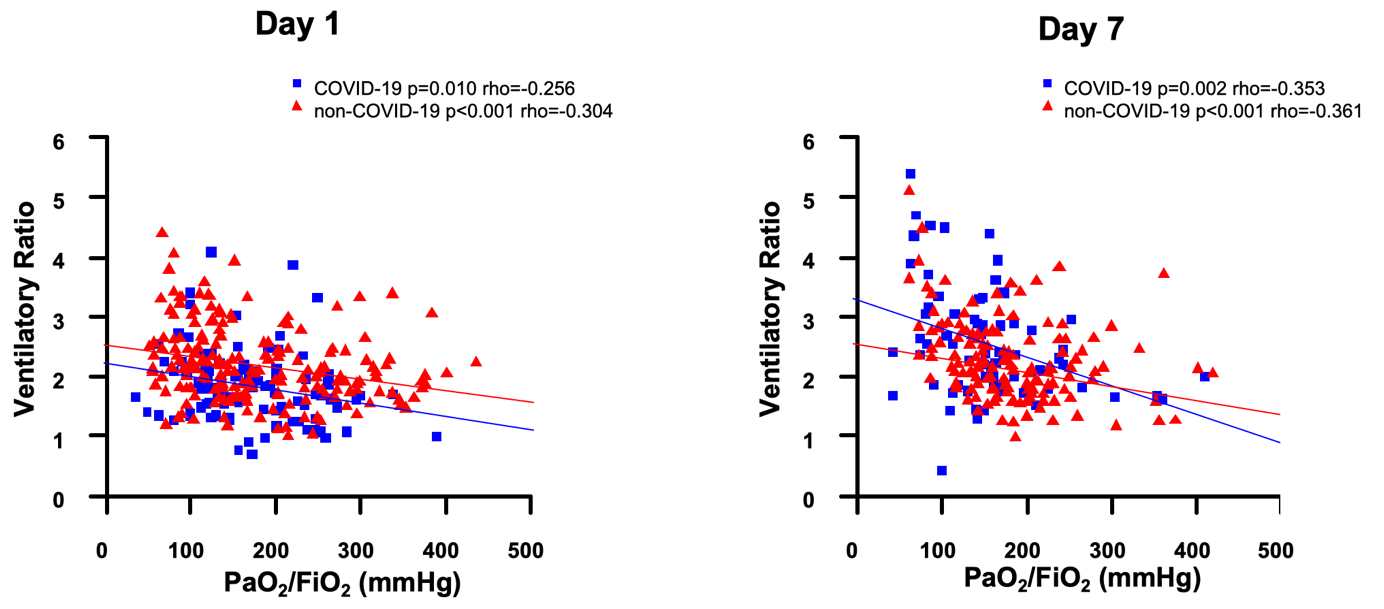
**

**Fig. S2** Respective correlations between the ratio of partial pressure of arterial oxygen (PaO_2_) over fraction of inspired oxygen (FiO_2_) and Ventilatory Ratio at day 1 (A) and day 7 (B) in the matched patients with COVID-19 and pulmonary non-COVID-19 associated ARDS.

**Fig. S3** Ventilatory Ratio at day 1, day 3 and day 7 in patients with COVID-19 associated Acute Respiratory Distress Syndrome with or without diagnosis of thrombo-embolic event. Boxplots display medians, 10th, 25th, 75th, and 90th percentiles.
